# Enhancing Cognitive Health in Aging through New Language Acquisition: Insights from Behavioral and Neuroimaging Studies

**DOI:** 10.1002/alz.094385

**Published:** 2025-01-09

**Authors:** Ladan Ghazi Saidi

**Affiliations:** ^1^ University of Nebraska Kearney, Kearney, NE USA

## Abstract

**Background:**

I will look at the impact of new language acquisition on cognitive performance and reserve in older adults, an area not extensively explored in contrast to the well‐documented cognitive benefits in lifelong bilinguals. Previous research has shown that lifelong bilingualism may enhance cognitive reserve, evidenced by better performance on specific tasks of executive function, reflecting superior cognitive control and interference management in aging (Van den Noort, et al., 2019). Drawing on the cognitive demands of language learning, which include executive functions such as inhibition, selection, and conflict management (Ghazi Saidi et al., 2013; 2017a; 2017b), we and others hypothesize that new language learning in older adults can boost these cognitive processes, which often decline with age (Albinet, et al., 2012; Lee & Chung, 2012).

**Method:**

In the past five years, we and others have conducted a series of behavioral and neuroimaging studies to address the gap in understanding the effects of language learning later in life, particularly as a potential non‐pharmaceutical intervention to enhance cognitive health in aging. In this presentation, I will review these studies and will present our new findings.

**Results:**

We are others bring evidence that Language learning in older adults can enhance cognitive control, as evidenced by improved performance on domain specific tasks such as the Stroop task, as well as global cognition and is associated with functional neuroplasticity in brain areas responsible for cognitive control (Kliesch, et al.,2018; Meltzer, et al., 2023; Nilsson, et al., 2021; Bubbico, et al., 2019; Wong, et al., 2019. These results suggest that new language acquisition in monolingual older adults may improve cognitive reserve and contributes to overall cognitive health, potentially slowing the progression of cognitive decline.

**Conclusion:**

Thus, learning a new language may represent an accessible and cost‐effective cognitive intervention in aging, though the effectiveness is influenced by the intensity and duration of the learning experience.

